# Soft Gels in Food Systems: Recent Advances, Applications, and Technological Innovations

**DOI:** 10.3390/gels11080667

**Published:** 2025-08-21

**Authors:** Manuela Machado, Eduardo Manuel Aguiar da Costa, Sara Silva

**Affiliations:** Universidade Católica Portuguesa, CBQF Centro de Biotecnologia e Química Fina-Laboratório Associado, Escola Superior de Biotecnologia, Rua Diogo Botelho 1327, 4169-005 Porto, Portugal; mmachado@ucp.pt (M.M.); snsilva@ucp.pt (S.S.)

**Keywords:** food structuring, soft gels, controlled release, regulatory compliance

## Abstract

Soft gels, such as hydrogels, organogels, aerogels, and bigels, represent versatile materials that are increasingly utilized within food systems to modify texture, regulate nutrient delivery, serve as fat substitutes, and enhance product shelf life. Their structural diversity and tunable properties enable targeted solutions for healthier, more sustainable, and consumer-centric products. This review provides a critical overview of recent advances in soft gel science, emphasizing industrial feasibility, regulatory compliance, and strategies to overcome commercialization barriers such as cost, scalability, and consumer acceptance. For each gel type, we compare functional performance with conventional structuring and encapsulation systems, highlighting cases where soft gels offer superior stability, bioactive protection, or caloric reduction. We also examine emerging applications, including gel-based frying media, 3D printing, and nano-enabled formulations, alongside potential risks related to long-term exposure and bioaccumulation. Regulatory frameworks across major jurisdictions are summarized, and sustainability considerations, from sourcing to life cycle impact, are discussed. By integrating technological innovation with safety, regulatory, and market perspectives, this review identifies key research priorities and practical pathways for translating soft gel technologies from laboratory concepts into commercially viable, health-driven food solutions.

## 1. Introduction

The modern food industry is increasingly driven by the demand for innovative ingredients that not only enhance the nutritional quality of products but also improve their functionality, stability, shelf life, and sensory appeal. In this context, soft gels, including hydrogels, organogels, aerogels, and bigels, have emerged as multifunctional structuring systems with broad applicability in food formulation, reformulation, and design. These materials offer promising solutions for creating foods with tailored textures, controlled bioactive release, and reduced fat or additive content, while also aligning with current trends in sustainable, health-driven innovation.

Soft gels are semi-solid systems formed by three-dimensional (3D) networks that immobilize either aqueous or lipid phases, resulting in matrices with distinct textural and rheological characteristics. Hydrogels, based on hydrophilic biopolymers such as alginate, gelatin, or carrageenan, are widely used for moisture retention, texture modification, and the encapsulation of hydrophilic bioactives [1]. Organogels (or oleogels), on the other hand, structure lipophilic phases using gelators like waxes or ethylcellulose, offering a promising alternative to conventional fats in spreads, pastries, and meat analogs [2]. Aerogels, known for their ultra-low density and high porosity, are gaining traction as delivery vehicles and active packaging materials [3]. More recently, bigels (hybrid systems that combine aqueous and lipid phases) have been proposed as advanced platforms for dual encapsulation and, subsequently, multifunctional delivery [4,5,6]. Despite substantial research progress, there is a lack of integrated reviews that critically examine the convergence of soft gel technologies with emerging food industry demands, particularly regarding precision structuring, fat replacement, bioactive delivery, and shelf-life enhancement. Moreover, novel production approaches, including 3D food printing and nanotechnology, have expanded the functional scope of soft gels by enabling precise architecture, personalized release kinetics, and improved mechanical performance [7,8,9,10]. However, these advances also introduce new challenges, such as the need to validate regulatory pathways, the toxicological assessment of novel gelators and nanostructures, and strategies for transparent labeling and consumer acceptance—areas that remain underexplored.

Therefore, this review addresses a knowledge gap by providing a comprehensive and critical overview of recent advances in soft gel systems within the food sector. It highlights their functional roles, materials, and mechanisms; innovative processing technologies; safety considerations; and regulatory landscapes. In doing so, it aims to clarify the scientific relevance and practical implications of soft gels as enabling platforms for the development of next-generation food products that are not only functional but also safer, more sustainable, and consumer-aligned.

## 2. Types of Soft Gels in the Food Industry

As previously mentioned, soft gels are a diverse group of structured materials characterized by their semi-solid consistency and the ability to immobilize liquid phases within 3D networks. In the food industry, the most relevant categories include hydrogels, organogels, aerogels, and bigels, each with distinct physicochemical properties and functional potentials. Their composition, ranging from hydrophilic to lipophilic systems, determines their application, whether for texturization, encapsulation, fat replacement, or stabilization. This section provides an overview of these gel types, highlighting their structure, formation mechanisms, and relevance to modern food design.

### 2.1. Hydrogels

**Figure 1:**
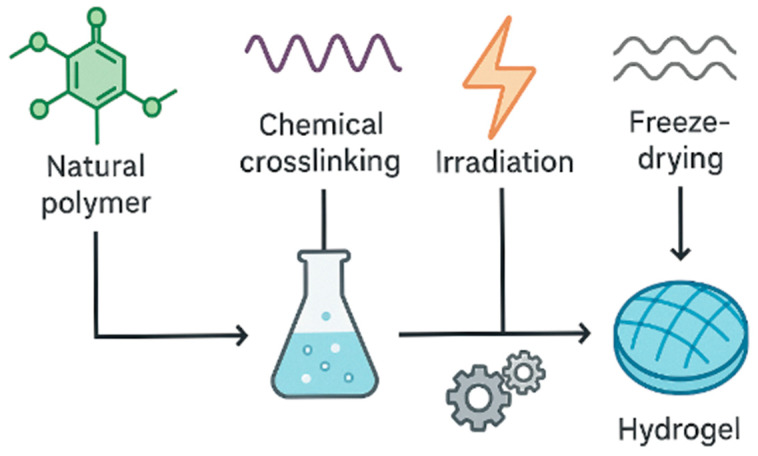
Overview of hydrogel production pathways. Created with Canva.com. (https://www.canva.com/) (accessed on 11 August 2025).

Hydrogels (Figure 1) are 3D polymeric networks formed by the crosslinking of hydrophilic polymer chains that are capable of containing relatively large amounts of water while maintaining structural integrity.

This results in a gel matrix that is soft, elastic, and highly hydrated, with adjustable physicochemical properties that are biocompatible and tunable to environmental stimuli. Thus, these structures have gathered attention for their potential use in several different fields, among which stands food science [11,12]. In fact, modern hydrogel technologies (beyond simple agar, gelatin, or starch gels) offer great versatility in terms of composition, structure, and function. Additionally, as they may also be designed to yield specific responses to key external stimuli (such as temperature, pH, or enzymatic activity), they allow for, as an example, the on-demand release of functional ingredients, a property not available in classical food gels. This adaptability not only opens avenues for creating health-promoting and functional foods but also aligns with trends in sustainable food design, such as the development of healthier and/or more nutrient-dense reformulated products [13,14,15,16].

The core of hydrogels is comprised of polymers that can either be physically or chemically crosslinked. In the first type—physically crosslinked hydrogels—non-covalent interactions (e.g., hydrogen bonds, electrostatic, and hydrophobic interactions) mediate interactions between the polymers, resulting in gels that are typically reversible and sensitive to environmental conditions such as fluctuations in pH, ionic strength, and temperature [15]. The second—chemically crosslinked hydrogels—involve the establishment of covalent bonds between polymer chains, which results in structures with improved mechanical stability and higher resistance to degradation under physiological conditions [17]. Overall, a wide variety of polymers can be used for hydrogel production and can be generally classified as natural, semi-synthetic, and synthetic. Natural polymers (e.g., alginate, pectin, gelatin, carrageenan, or chitosan) are particularly interesting when considering the development of hydrogels for food applications, given their non-toxicity, biodegradability, inherent biocompatibility, and the consumer’s overall preference for naturally occurring ingredients [18,19].

Derived from brown algae, alginate is an example of a polymer widely used in the food industry. It is an anionic polysaccharide copolymer composed of β-D-mannuronic acid and α-L-guluronic acid subunits that form gels via ionic crosslinking with divalent cations (e.g., Ca^2+^). Also with significant use by the food industry is pectin, a plant-derived anionic polysaccharide composed primarily of galacturonic acid. Depending on the esterification level, it can form hydrogels either via ionic interactions (low-methoxy pectins) or sugar–acid interactions (high-methoxy pectins), characteristics that make pectins a versatile gelling agent in both low- and high-sugar food systems [20]. Lastly, but also widely used, is gelatin. It is obtained via the partial hydrolysis of collagen and forms physically crosslinked hydrogels through a triple-helix formation at temperatures below 35 °C. Its intrinsic thermoreversibility and gelling strength make it a widely used protein-based hydrogelator typically used in desserts, yogurts, and gummy candies [21]. Another widely used hydrocolloid is gellan gum, an extracellular polysaccharide produced by *Sphingomonas elodea*. It forms strong, thermally stable gels via ionic crosslinking with monovalent or divalent cations [22,23,24]. Gellan gum is valued for its high clarity, heat stability, and ability to gel at very low concentrations, making it particularly suitable for suspending particulates in beverages, stabilizing plant-based desserts, and creating firm, elastic textures in low-calorie formulations.

Protein-based hydrogels also represent a versatile class of food-structuring agents. Among dairy proteins, whey protein isolate (WPI), containing more than 90% protein, produces transparent, fine-stranded gels with high gel strength and minimal lactose—making it ideal for high-protein clear beverages, gels, and encapsulation matrices. In contrast, whey protein concentrate (WPC), which contains ~35–80% protein along with residual lactose and lipids, yields softer, more opaque gels that contribute to creamy textures in yogurts, dairy desserts, and spreads [25,26,27]. Selection between WPI and WPC allows food technologists to fine-tune a gel’s firmness, transparency, and nutritional profile depending on the target product. Other proteins, such as soy proteins, can also be used to produce protein-based hydrogels with tailored gel strength, porosity, and water-holding capacity [28,29]. A more detailed list of the different types of polymers used to produce hydrogels can be found in Table 1.

Regarding hydrogels, from a food industry perspective, their use as mere gelling agents is a reduction of their true potential. In fact, these scaffolds have evolved into a multifunctional platform capable of addressing an array of different challenges like the development of novel fat replacers, delivery systems for nutrients, flavor or bioactive ingredients, and texture modifiers, and can even be used as a part of smart packaging solutions [12,60]. An example is the use of hydrogel-based fat mimetics that were developed to replicate the mouthfeel and lubrication properties of fats (without compromising flavor or texture) in products aiming at reduced fat content, such as low-fat spreads, dressings, and dairy alternatives [61]. These hydrogel systems are often composed of biopolymer-based emulsions or oleogels that trap oil within water-rich gel matrixes, resulting in a reduction of the overall fat content (and thus reducing overall caloric content) while maintaining the product’s characteristics. Another important example of the use of hydrogels beyond their function as gelling agents is their use as an encapsulation system that allows for the controlled release of bioactive ingredients. This is particularly interesting as this strategy may allow for the protection of sensitive compounds like vitamins and antioxidants, protecting them during food processing, storage, and even gastrointestinal digestion. Namely, Hassan et al. (2022) used alginate and chitosan hydrogels to encapsulate phenolic compounds to not only enhance their stability but also modulate their release, targeting specific sections of the digestive tract [62]. When compared with other traditional encapsulation methods, like spray- or freeze-drying, hydrogels frequently outperform them in terms of sustained release and the preservation of compound bioactivity [60].

### 2.2. Organogel

**Figure 2:**
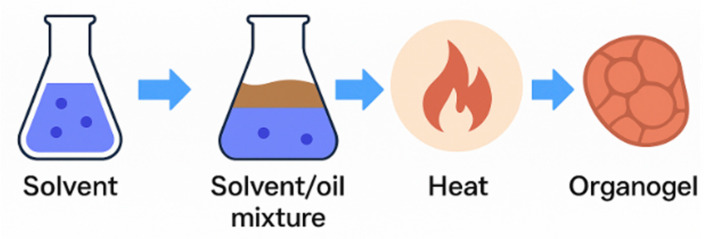
Simplified process for organogel formation. Created with Canva.com. (https://www.canva.com/) (accessed on 11 August 2025).

Organogels (Figure 2), also referred to as oleogels when oil serves as the continuous phase, are semi-solid systems composed of an organic liquid immobilized within a 3D network created by gelator molecules. These gelator molecules range from low-molecular-weight compounds (e.g., waxes, sterols, and lecithin) to larger polymeric or crystalline structures [38,63].

One of the fundamental mechanisms behind organogel formation implies thermal gelation and subsequent self-assembly. It implies the heating of an oil–gelator mixture followed by its cooling, which allows for organogel formation via the establishment of non-covalent interactions like van der Waals forces, hydrogen bonding, π–π stacking, or the crystallization of gelator molecules into fibrous/crystalline networks [64]. Tang et al. [65] reported an example of this type of organogel using stigmasterol as a gelator. These authors reported that for concentrations as low as 2%, this phytosterol could achieve >99% oil binding capacity, resulting in the formation of rod-like crystalline fibers (formed through self-assembly and hydrogen bonding) [65]. Organogels may also be polymer-based, being formed through covalent cross-linking and subsequent network formation that results in more robust and less thermally reversible gel networks suitable for structured oils [64].

The appeal of organogels for food industry use stems from their ability to replicate the functional roles of conventional fats (namely by imparting structure, creaminess, lubrication, and flavor retention) while also allowing for the replacement of saturated fats with healthier unsaturated oil matrices. Previous reviews have highlighted how oleogels may match (or even surpass) the performance of traditional fats in terms of texture and thermal stability while simultaneously yielding products with an improved nutritional value, even possibly supporting clean-label usage and being more sustainable innovations [66,67,68,69]. An example of this is oleogels. Structured using natural waxes or lecithin as gelators, they can be tailored to mimic the texture and melting profile of animal fats, reducing their use in products like pâtés, meat batters, and margarine-like products [42,43,70,71]. However, when considering their use as far as replacement in foods, it is important to keep in mind that while there is an improvement in nutritional profile, other challenges related to the sensory characteristics, processing costs, and oxidative stability of the products may arise [67].

Organogelators have a large structural diversity, ranging from natural waxes (e.g., carnauba or rice bran wax) and ethylcellulose to sterol-based systems that use β-sitosterol or γ-oryzanol. This variety permits customization of organogels’ rheological and textural properties like firmness, spreadability, or mouth-coating, and, subsequently, makes them suitable for a wide range of food applications. Moreover, it also makes them suitable for the development of delivery systems for lipophilic bioactives (e.g., fat-soluble vitamins or phytosterols), facilitating their incorporation into functional foods [2,39,61,63,64,72].

Despite their promising applications, organogels face several barriers to their wider adoption by the food industry. These barriers include (1) the need for edible, Generally Recognized as Safe (GRAS)-listed gelators; (2) a better understanding of the interactions and reactions that may occur under different food processing stresses; (3) lower oxidative stability of unsaturated oils; (4) the need for a cost-effective scaling of the organogel producing processes; and (5) achieving consumer-acceptable sensory profiles [2,67].

### 2.3. Aerogels

**Figure 3:**
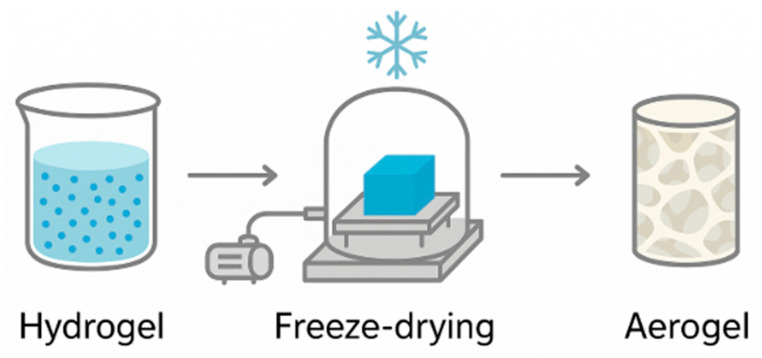
Schematic representation of hydrogel-to-aerogel conversion via freeze-drying. Created with Canva.com. https://www.canva.com/ (accessed on 11 August 2025).

Aerogels (Figure 3) are an emerging class of porous, lightweight materials characterized by high surface area and open pore structures, typically containing more than 90% air by volume. Originally developed for aerospace and thermal insulation, their unique physical and chemical attributes have attracted growing interest in food science and technology. Their low density, high porosity, and large specific surface area (commonly 100–600 m^2^/g) make them promising candidates as bioactive carriers, texture modifiers, fat replacers, and functional components in smart packaging. Unlike freeze-dried matrices, aerogels are produced using specialized drying methods—most notably, supercritical CO_2_ drying—which preserve the gel’s 3D network and prevent pore collapse [73,74].

The fabrication of an aerogel typically involves three main stages: gelation, solvent exchange, and drying. Gelation occurs through chemical or physical crosslinking of a polymer network, often using biopolymers such as cellulose, alginate, pectin, starch, or gelatin. Following gelation, the solvent (commonly water) is replaced with a low-surface-tension organic solvent such as ethanol or acetone to reduce capillary forces and minimize structural damage. Finally, drying is performed, with supercritical CO_2_ drying considered the most effective for retaining matrix structure, as it avoids the liquid–gas phase transition and associated network collapse. Freeze-drying provides an alternative, sublimating the frozen solvent directly, although it often yields slightly lower surface areas and more regular pore structures [75,76,77].

Food-grade aerogels are predominantly derived from renewable biopolymers, with polysaccharides like nanocellulose, starch, and pectin being of particular interest due to their biodegradability and good gelling properties. Considering the current sustainability trends and the consumer’s demand for more sustainable products, their use also aligns with these concerns as they can be readily sourced from industrial byproducts. In line with this, Gaggero et al. [73] demonstrated that spent coffee and fruit pomace could be successfully used to produce aerogels with tunable pore characteristics, linking food waste valorization with advanced material applications [73]. As an alternative, proteins, particularly whey and gelatin, may also be used to produce edible aerogels with promising loading and release properties for both bioactive ingredients and micronutrients [3,74,78].

The multifunctionality of aerogels underpins their potential in food applications. Their internal porosity enables loading with a wide range of compounds—health-promoting bioactives, flavors, nutrients, antioxidants, or functional lipids—while offering protection against oxidation and thermal degradation. Compared with conventional encapsulation methods (e.g., spray drying or emulsions), aerogels often provide more controlled release profiles and the improved stability of sensitive ingredients [3,79,80,81]. This makes them particularly promising for fortifying beverages, dairy products, and baked goods with unstable or bitter bioactives, such as polyphenols, omega-3 fatty acids, or probiotics. Oil-loaded edible aerogels have also been proposed as fat replacers, capable of mimicking the creamy mouthfeel and rheology of conventional fats while reducing caloric content and enabling gradual lipid release during digestion, potentially enhancing satiety and nutritional profiles [3,82].

Despite their promise, several challenges limit the large-scale adoption of aerogels in food systems. The most common drying method, supercritical CO_2_ drying, is energy-intensive and costly. Moreover, the regulatory approval of some edible biopolymer-based aerogels remains incomplete, especially for novel formulations intended for ingestion or direct food contact, where safety, migration potential, and consumer perception must be addressed. Finally, the mechanical fragility of low-density aerogels makes them prone to collapse during processing and handling [3,52,83].

Recent technological advances are beginning to address these barriers. To reduce costs, researchers are exploring hybrid drying methods such as ambient pressure drying combined with surface modification that partially preserves pore structures while lowering equipment and energy demands [84,85,86]. Advances in freeze-drying protocols, including the use of cryoprotectants and templating agents, also improve structural retention at a lower cost. On the regulatory front, the use of well-established, GRAS-status biopolymers (e.g., alginate, pectin, and cellulose) and sourcing from recognized food-grade byproducts can streamline approval processes [87,88,89]. Preemptive safety dossiers, migration testing, and collaborative engagement with food safety authorities are strategies that may accelerate market authorization. To address mechanical fragility, innovations include reinforcement with nanocellulose fibrils, blending with protein networks to improve flexibility, and incorporating crosslinking agents that enhance resilience without compromising edibility. Packaging design can also play a role, with the protective encapsulation of aerogel-based inclusions in intermediate food components to shield them from mechanical stress. Together, these strategies illustrate a clear research trajectory toward more robust, cost-effective, and regulation-compliant aerogels, paving the way for their wider integration into functional and sustainable food products.

### 2.4. Bigels

**Figure 4:**
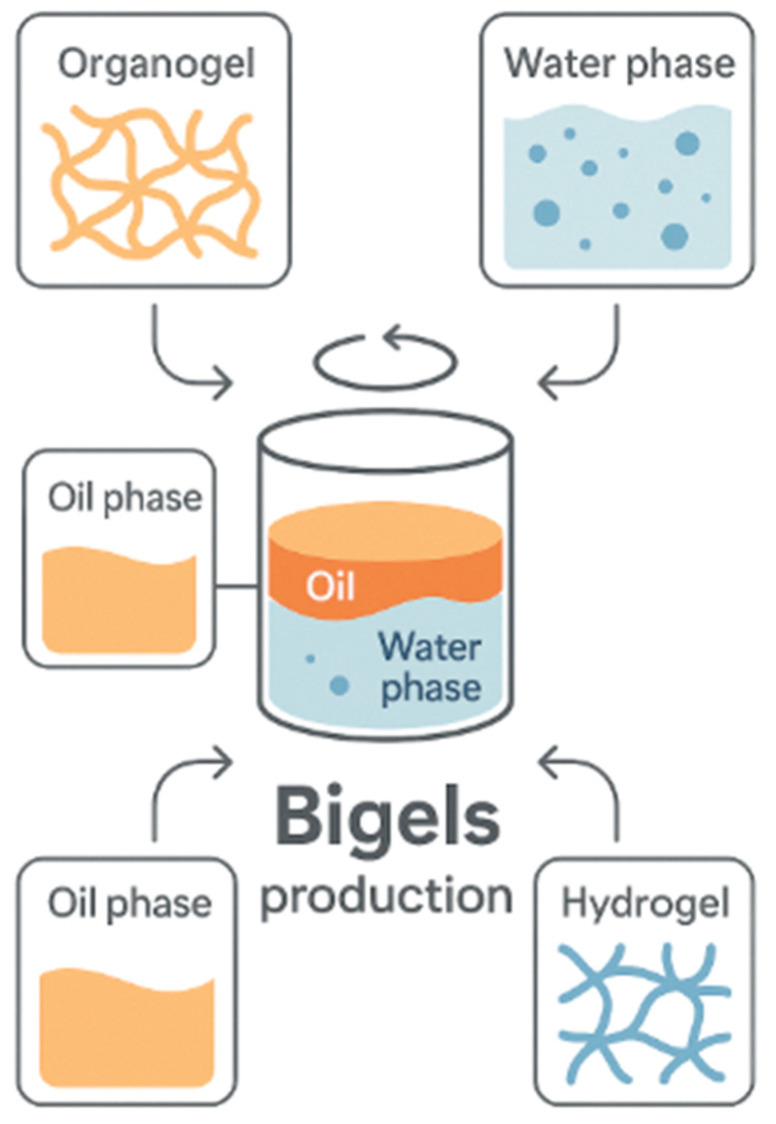
Schematic representation of bigel production from oil and water phases. Created with Canva.com. https://www.canva.com/ (accessed on 11 August 2025).

Bigels (Figure 4) are emerging as versatile biphasic soft gel systems, and are composed of an interpenetrated or co-continuous network formed by a hydrogel phase and an organogel (or oleogel) phase.

This hybrid structure results from the physical combination of aqueous and lipid domains, stabilized through thermodynamic compatibility, emulsification techniques, and the use of emulsifiers or stabilizers. Unlike single-phase gels, bigels enable the simultaneous delivery and structuring of both hydrophilic and lipophilic components within a single matrix, granting them a unique functional advantage in complex food systems [4,6,90,91]. Bigel formation typically involves the independent preparation of the hydrogel and organogel phases, followed by a homogenization step where both systems are blended under controlled shear, temperature, and pH conditions. The gelators involved can range from common food-grade biopolymers (e.g., gelatin, alginate, carrageenan) in the hydrogel phase to lipid-structuring agents like beeswax, sunflower wax, or lecithin in the organogel phase [57,58,92,93]. The resulting structure is influenced by several factors, including the ratio between the two phases, the viscoelastic properties of each component, and the interfacial interactions that occur upon mixing [94].

Several studies have demonstrated the potential of bigels in food applications, particularly as systems for fat replacement, controlled release, and functional emulsion stabilization. One of the most significant applications is the efficient delivery of bioactive compounds. Numerous studies have shown improvements in the delivery of both lipophilic and hydrophilic bioactives, including vitamins, phytosterols, phenolic compounds, and fatty acids [51,52,53,54,55,56,57,58].

From a mechanistic perspective, bigels benefit from the synergistic interaction between the hydrogel’s water-retention capacity and the organogel’s lipid structuring, resulting in systems that are more thermally stable, structurally resilient, and multifunctional than their monophase counterparts. Their modularity and tunability make them suitable for the design of personalized nutritional products, functional food formulations, and targeted delivery vehicles for both hydrophilic (e.g., vitamin C, polyphenols) and lipophilic compounds (e.g., vitamin D, phytosterols).

However, bigels face several technological and commercial challenges that limit their broader adoption by the food industry. These limitations include maintaining phase compatibility to prevent separation, selecting gelators and emulsifiers deemed GRAS- or EFSA-approved, addressing oxidative stability issues associated with unsaturated lipid fractions, economically scaling up production processes, and achieving sensory properties that meet consumer preferences in different food categories.

Nonetheless, bigels hold considerable promise as structuring agents and multifunctional delivery systems, particularly in reformulated or health-oriented food products. Future research will likely focus on improving their scalability, refining their sensory characteristics, and exploring new bioactive incorporation strategies—paving the way for novel applications in both mainstream and functional food sectors [95].

## 3. Functional Roles of Soft Gels in the Food Industry

Soft gels have emerged as multifunctional materials in food systems, offering innovative solutions to modulate the structure, deliver bioactive compounds, reduce calorie content, and extend shelf life. Their functional performance is governed by the physicochemical properties of their matrices, which can be tailored through material composition, network architecture, and processing techniques. The following sections explore how these materials fulfill key technological roles, discussing findings from the recent literature and how they relate to current hypotheses in food structuring and delivery science.

### 3.1. Texture Modification

Hydrogels and bigels are extensively used to modify the textural attributes of food products by influencing viscosity, elasticity, firmness, and mouthfeel. Crosslinked networks based on polysaccharides (e.g., alginate, carrageenan) or proteins (e.g., gelatin, whey protein) can form viscoelastic gels with tailored mechanical properties, with several studies showing that varying the gelator concentration and crosslinking degree allows for fine control over the rheological behavior of products like yogurts, sauces, creams, and desserts [96,97,98,99].

Advanced designs include dual-network hydrogels, freeze–thaw-induced gels, and composite systems reinforced with nanocellulose or complementary biopolymers, and these can enhance structural resilience during processing, storage, and temperature fluctuations. This capacity for structural customization supports the hypothesis that soft gels can mimic or replace complex food matrices without compromising sensory quality. Bigels, which combine oil and water phases, can create creamy, spreadable textures desirable in dairy analogs, plant-based spreads, and low-fat reformulations [100,101,102,103,104,105,106,107].

Compared to traditional texture modifiers such as starch pastes, pectin gels, or fat-based emulsions, soft gels offer several notable advantages: (i) greater tunability of viscoelastic parameters without relying solely on high solid content, (ii) improved stability under shear and thermal stress due to tailored crosslinking, and (iii) the ability to integrate additional functionalities such as encapsulated bioactives or responsive release triggers. However, challenges remain in scaling certain advanced gel systems, particularly those requiring costly precursors or specialized processing—and in fully matching the flavor release and mouth-coating characteristics of high-fat traditional systems. This indicates that while soft gels can outperform conventional approaches in adaptability and multifunctionality, their adoption often depends on balancing sensory performance with processing and cost constraints.

### 3.2. Gels as Deep Frying Media

Recent studies have explored the use of oleogels and aerogels as innovative frying media to reduce oil uptake in fried foods while maintaining desirable sensory qualities. Traditional frying methods often lead to excessive fat absorption, which increases caloric content and promotes lipid oxidation. In contrast, gel-based frying systems can modulate oil transport, act as structural barriers, and deliver healthier lipid profiles.

Oleogels, structured with gelators such as waxes, ethylcellulose, or phytosterols, have been shown to substitute conventional frying oils effectively. Sunflower oil–soybean wax oleogels achieved up to 37.8% less oil absorption in doughnuts compared to sunflower oil while improving oxidative stability indicators such as peroxide value, free fatty acids, and p-anisidine value [108]. Similar effects were observed with sunflower oil–candelilla wax systems, which reduced oil uptake in fried chicken and battered vegetables while maintaining texture and flavor [109]. In another example, rice bran wax–canola oil oleogels used for frying fish fillets not only reduced the final oil content but also limited the formation of harmful polar compounds while enhancing the aromatic profile [110]. A recent review confirmed that oleogels consistently decrease fat absorption without compromising product sensory properties [111].

Aerogels, particularly those derived from cellulose or starch, have been proposed as frying aids or pre-frying coatings. Their ultra-porous structure and high surface area allow for moisture release while reducing oil penetration. Although direct studies on aerogels in frying are limited, research on hydrocolloid-based coatings such as carrageenan and xanthan gum has shown 25–57% reductions in oil uptake in fried snacks [112], suggesting that aerogel coatings could achieve comparable results. Such coatings also preserve crispness and reduce oxidation, improving the overall quality of fried products. Despite these promising findings, challenges remain in maintaining gel structural integrity at frying temperatures, optimizing gelator concentration, and ensuring uniform application. Additionally, the sensory implications of these gels, particularly on mouthfeel and flavor release, require further investigation to ensure consumer acceptance. Nevertheless, gel-based frying systems represent a promising frontier for reformulating fried foods, enabling fat reduction, healthier lipid profiles, and potential for functional compound delivery, all in alignment with public health goals and clean-label innovation.

### 3.3. Encapsulation and Controlled Release

One of the most explored functions of soft gels in food technology is the encapsulation and controlled delivery of functional compounds such as vitamins, polyphenols, antioxidants, and probiotics. Hydrogels have demonstrated excellent capabilities in protecting hydrophilic compounds from thermal, oxidative, and enzymatic degradation. Their swelling-responsive networks allow for a controlled release mechanism influenced by pH, temperature, or ionic strength, as reported by Li et al. [60]. Organogels and oleogel-based systems, in turn, are more suitable for lipophilic compounds (e.g., omega-3, β-carotene), as they form structured lipid matrices that release bioactives during lipolysis [93,113,114]. More recently, bigels (hybrid systems combining hydrogel and organogel networks) have shown promise for the co-encapsulation of both hydrophilic and lipophilic ingredients. These systems align with the working hypothesis that biphasic gels can enhance bioavailability and enable targeted nutrient delivery across the gastrointestinal tract [58,59,95,115,116,117]. Aerogels, with their highly porous structure, provide an alternative route for high-efficiency encapsulation and sustained release via adsorption and diffusion-controlled mechanisms, further supporting the integration of soft gels in functional foods and nutraceuticals.

When compared with traditional delivery systems such as spray-dried powders, simple emulsions, or lipid capsules, soft gels frequently demonstrate superior protection against environmental and processing stresses [118,119]. Their three-dimensional networks can physically isolate bioactives from degradation factors while offering more precise control over release profiles. This is something that is often lacking in conventional systems, where release is largely diffusion-driven and less responsive to physiological triggers. Additionally, bigels and aerogels enable the simultaneous loading of compounds with very different solubility profiles, a capability rarely achievable with traditional single-phase carriers [4].

Nonetheless, traditional systems retain certain advantages: spray-drying remains significantly more cost-effective at a large scale, and simple emulsions often require less specialized equipment or expertise. Moreover, in some applications, such as highly hydrophobic compounds, lipid capsules can achieve comparable stability at lower formulation complexity. Therefore, while soft gels provide a more versatile and targeted platform for delivery, their commercial adoption will depend on balancing these functional benefits against economic feasibility, formulation of shelf-life stability, and compatibility with existing processing lines.

### 3.4. Fat Replacement and Caloric Reduction

Organogels and bigels have gained increasing attention as fat replacers in processed foods, offering structural and sensory properties similar to those of traditional fats but with significantly lower caloric density [66]. By structuring liquid oils into semi-solid matrices using food-grade gelators (such as sunflower wax, ethylcellulose, or glycerol monostearate), researchers have created organogels that can replace saturated and trans fats in products like margarine, spreads, baked goods, and meat analogs.

Research indicates that substituting conventional fats with alternative formulations can result in up to a 50% reduction in saturated fat content while preserving satisfactory textural and sensory attributes. Additionally, the thermomechanical properties of these systems can be optimized to replicate the plasticity and melting characteristics of animal fats, which is essential for reformulated food products. Further studies have highlighted the potential of bigels to enhance the nutritional profile of dairy items by increasing polyunsaturated fatty acid levels [115,117,120,121,122].

Bigels further extend these benefits by providing a balanced combination of aqueous and lipid phases, thereby improving nutritional value without diminishing product stability or mouthfeel. Collectively, these findings substantiate the proposition that gel-structured fats represent viable, health-promoting alternatives capable of maintaining consumer acceptability.

### 3.5. Stabilization and Shelf-Life Enhancement

Soft gels also contribute to food stability by mitigating microbial growth, oxidative degradation, and moisture loss [95]. Hydrogels, for example, are widely employed as edible coatings or films that act as physical barriers while delivering active compounds such as essential oils or antioxidants. These coatings can modulate water activity and oxygen permeability, two key parameters in spoilage control, while reducing the need for synthetic preservatives. Aerogels, with their ultra-porous structure and high surface area, are particularly suitable for incorporating volatile or sensitive bioactives. Studies have shown that aerogels loaded with phenolic compounds or essential oils can release these agents gradually, sustaining antimicrobial and antioxidant effects over extended storage [74,123]. Bigels are increasingly applied in functional coatings or fillings that encapsulate preservatives, flavor modulators, or sensory enhancers, protecting them within the gel matrix and enabling controlled release under refrigerated or ambient conditions. This not only improves product stability but also aligns with cleaner-label formulations by reducing direct additive incorporation into the bulk food.

Beyond preservation, soft gels emerge as functional components in intelligent and active packaging systems. Their tunable mechanical and chemical properties allow them to serve as carriers for sensing elements that respond to environmental changes. Hydrogel-based sensors functionalized with anthocyanins have been developed as pH and total volatile basic nitrogen (TVB-N) indicators for the real-time monitoring of pork, shrimp, and fish freshness under commercial storage conditions [124,125]. Similarly, polydiacetylene (PDA) hydrogel beads and PDA/ZnO nanocomposite gels exhibit distinct colorimetric shifts in the presence of biogenic amines released during meat spoilage, providing both specificity and visual clarity [126]. Other studies have demonstrated that reinforcing pH-responsive hydrogels with nanocellulose or nanofibers not only improves their sensitivity to spoilage gases but also enhances their mechanical robustness, a critical factor for integration into commercial packaging lines [127]. Recent advances include oxygen-responsive gels with immobilized dyes or nanoparticles that detect oxidation in packaged foods, as well as moisture-sensitive gels that swell or change color when humidity changes indicate a package seal failure or water intrusion [128,129,130,131,132,133,134,135]. Additionally, gel-based sensing layers can be designed to monitor changes in barrier properties by tracking the rate of oxygen or moisture transmission through packaging films, providing early detection of material fatigue or degradation during storage [131,136,137]. These sensor-integrated gels can be incorporated into labels, coatings, or inserts that detect pH changes, amine release, oxygen ingress, CO_2_ buildup, or moisture penetration—early indicators of product degradation or package failure [132,133,136,137].

Integrating sensing functions into gel-based packaging facilitates more precise shelf-life estimation and may help reduce food waste [135]. In addition to their preservation abilities, soft gels can modulate texture, deliver nutrients in a targeted manner, serve as fat replacers, offer antimicrobial properties, extend shelf life, and enable real-time quality monitoring. These features position soft gels as platforms for innovation, health-related applications, and sustainable packaging.

## 4. Innovative Technologies in Soft Gel Production

The advancement of food-grade soft gels has been significantly enhanced by the integration of cutting-edge technologies that allow for precise control of their structure, function, and bioactivity. Two emerging approaches, 3D printing and nanotechnology, have been transforming the way soft gels are formulated, customized, and delivered in food systems.

### 4.1. Three-Dimensional Printing of Soft Gels

Three-dimensional printing is transforming the design and structuring of food matrices by enabling the precise, layer-by-layer deposition of gel-based materials, often referred to as “food inks.” In the context of soft gels, this technology facilitates the fabrication of customized food structures using hydrogels, bigels, and oleogels, which can be engineered for specific textures, nutrient compositions, and controlled release functionalities. Recent studies have shown that food-grade gels based on starch, gelatin, carrageenan, pectin, and whey protein exhibit excellent printability, which is characterized by suitable viscoelastic behavior, shear-thinning properties, and post-printing structural stability. These gels can be enriched with bioactive compounds, flavors, or nutrients and extruded into intricate 3D geometries that enhance visual and sensory appeal while also modulating gastric disintegration and nutrient absorption kinetics [10,138,139].

This approach aligns with the emerging hypothesis that personalized nutrition and functional food innovation can be achieved through modular, digital food structuring [138,139,140,141]. Printing parameters such as infill density, pore architecture, and layer orientation can be optimized to control the release kinetics of encapsulated actives, adjust mechanical response during mastication, and influence satiety or mouthfeel. Moreover, 3D printing enables the creation of tailored foods for specific populations—such as elderly individuals, children, or patients with dysphagia—by producing soft, easy-to-swallow textures that meet precise nutritional and physiological requirements [138].

Despite these advances, the integration of 3D printing and nanotechnology with soft gels remains underexplored in several critical areas. The long-term safety and regulatory implications of incorporating nanostructured gelators or nano-enabled delivery systems into printed foods are not yet fully understood, particularly with respect to migration, bioaccumulation, and chronic exposure effects [142]. The stability and performance of nano-functionalized gels under real-world processing, storage, and gastrointestinal conditions require deeper investigation, as do the scalability and cost-effectiveness of such hybrid systems for industrial production. Furthermore, the interactions between nanoscale components and the mechanical and rheological behavior of printed gels are still poorly characterized, limiting the ability to predict performance and consumer perception [138,141,142]. Addressing these knowledge gaps will be essential for advancing the safe, efficient, and commercially viable application of 3D-printed, nano-enabled soft gels in the food sector.

### 4.2. Nanotechnology Approaches

Nanotechnology has emerged as a transformative approach for soft gel functional performance enhancement in food applications. By incorporating nanoscale components such as nanocellulose fibrils, nanoemulsions, solid lipid nanoparticles, or polymeric nanocarriers into hydrogel, organogel, or bigel matrices, it is possible to significantly improve their mechanical integrity, stimuli response, and precision in active compound delivery [8]. Nanocellulose, in particular, provides reinforcement through strong hydrogen bonding and high aspect ratio entanglement, producing gels with greater elasticity, compressive strength, and resistance to syneresis. Aerogels reinforced with nanocellulose exhibit ultra-high porosity, high specific surface area, and exceptional loading capacity, while enabling tunable release via diffusion, swelling dynamics, or enzymatic triggers [74,143,144]. Nano-enabled gels offer distinct advantages for encapsulating fragile and poorly soluble ingredients, including probiotics, omega-3 fatty acids, polyphenols, and lipid-soluble vitamins by protecting them from oxidation, thermal degradation, and gastrointestinal stress. For example, nanoemulsion droplets embedded within hydrogel networks can improve the bioaccessibility of lipophilic bioactives, while nanoparticle–hydrogel composites can achieve multi-phase release profiles adapted to specific GI transit stages.

Beyond passive protection, nanostructured gels are increasingly engineered as “smart systems” capable of responding to environmental cues such as pH shifts, ionic strength changes, temperature fluctuations, or enzymatic activity [7,13]. Such responsiveness enables targeted release at specific sites in the digestive tract, enhancing both efficacy and dose control. In some designs, nanoparticle carriers are functionalized with ligands or biorecognition elements, allowing for selective interactions with target biomolecules or microbial populations.

When compared with conventional gel systems lacking nanoscale reinforcement, nano-enabled soft gels typically offer superior mechanical stability, controlled degradation rates, and higher encapsulation efficiencies, though they may require more complex formulation strategies and stricter regulatory oversight [145,146]. The introduction of engineered nanomaterials into food systems raises the need for thorough safety assessment, including the characterization of nanoparticle migration, bioaccumulation potential, and long-term exposure effects [147,148].

Overall, nanotechnology integration within soft gel matrices represents a powerful platform for bridging the gap between structural robustness and advanced functionality [149]. By enabling both improved physical stability and programmable release, nano-enabled gels expand the design space for next-generation functional foods, nutraceuticals, and even active packaging solutions.

## 5. Regulatory and Safety Aspects

Soft gel incorporation into food systems introduces regulatory and safety considerations that are fundamental to their responsible development and successful commercialization. Regulatory frameworks across major jurisdictions—here defined as distinct legal or regulatory regions with their own binding statutes and enforcement authorities—set the permissible materials, maximum usage levels, and safety evaluation protocols for both gel matrices and the compounds they encapsulate. In addition to long-established benchmark systems, such as those of the European Union (EU), the United States Food and Drug Administration (FDA), Health Canada, Food Standards Australia New Zealand (FSANZ), and Japan’s Ministry of Health, Labor, and Welfare (MHLW), other major markets, including India, China, and Brazil, maintain comprehensive approval pathways for food additives and food-contact materials.

In the EU, food additives are governed by Regulation (EC) No. 1333/2008 and food-contact materials are governed by Regulation (EU) No. 10/2011, with safety assessments conducted by the European Food Safety Authority (EFSA) 10 [150,151]. In the USA, gel-forming agents and structuring additives must either be listed as Generally Recognized as Safe (GRAS) under 21 Code of Federal Regulations (CFR) Parts 170–186 or undergo the Food Contact Notification (FCN) process [152,153]. Comparable systems exist in Canada through the Lists of Permitted Food Additives, in Australia/New Zealand under the Food Standards Australia New Zealand (FSANZ)—Food Standards Code, and in Japan through the Food Sanitation Act and the Positive List System for food-contact materials. India regulates through the Food Safety and Standards (Food Products Standards and Food Additives) Regulations, 2011, and the Food Safety and Standards (Packaging) Regulations, 2018, via the Food Safety and Standards Authority of India (FSSAI) [154,155,156,157,158,159,160]. In China, the National Food Safety Standard for Uses of Food Additives (GB 2760 standard) governs additive use, while the GB 4806 series cover specific food-contact material categories under the National Health Commission (NHC). Brazil’s Agência Nacional de Vigilância Sanitária (ANVISA) oversees additive approvals and food-contact materials through RDC frameworks aligned with MERCOSUR resolutions [161,162,163,164].

Many gel-forming agents used in soft gels—such as alginate, pectin, gelatin, carrageenan, xanthan gum, and agar—have a long history of safe use, with broad regulatory acceptance based on their biocompatibility, biodegradability, and functional versatility. In organogels and oleogels, structuring agents such as sunflower wax, beeswax, glycerol monostearate, rice bran wax, and ethyl cellulose are approved in multiple jurisdictions, all subject to toxicological limits and migration testing. However, the emergence of novel gelators, nanostructured systems, and advanced manufacturing methods such as 3D printing introduces new regulatory challenges. While certain edible polymers are approved for 3D printing of food products, all process-related components—including printing nozzles, supports, and any auxiliary materials—must comply with strict migration limits and compositional requirements under food-contact legislation to prevent contamination.

To facilitate the navigation of the evolving global regulatory environment, Table 2 provides an overview of the principal approval frameworks, essential safety assessment procedures, and authoritative references for major jurisdictions. The table underscores both pre-market evaluation and post-market monitoring requirements.

Compliance with these frameworks extends beyond the composition of the gel matrix to the encapsulated compounds themselves. Soft gels, particularly hydrogels and aerogels, are highly effective at preserving the bioactivity of sensitive compounds such as vitamins, polyphenols, and probiotics by mitigating degradation pathways, including oxidation, hydrolysis, and enzymatic breakdown [18,165]. The incorporation of nanomaterials, reactive emulsifiers, or stimuli-responsive polymers can further enhance delivery performance, enabling controlled release and improved bioavailability [166,167]. However, such advancements also require more comprehensive safety evaluations. Critical assessments include simulated gastrointestinal digestion studies to identify degradation products, chronic exposure testing to detect cumulative or delayed effects, bioaccumulation risk analysis for slowly degradable gelators, and migration testing for processing-related residues. Given the potential for long-term or low-level risks to emerge only after extended use, continuous post-market surveillance, adverse event reporting, and regular compliance audits are indispensable [168].

Beyond technical safety, consumer acceptance is a decisive factor in the successful adoption of soft gel technologies [169]. Transparent and accurate labeling, such as specifying “vitamin D encapsulated in alginate microbeads to support bone health”, can help build trust by communicating both the nature and the function of encapsulated ingredients. Nonetheless, labeling alone may not be sufficient to address skepticism toward “engineered” or “nano-enabled” components. Broader engagement strategies are therefore essential. These may include public education campaigns that explain the scientific basis and safety validation of soft gels, third-party certifications to verify product claims, open access to toxicological and regulatory data, collaboration with healthcare professionals to disseminate evidence-based information, and the use of interactive tools such as QR codes or augmented reality demonstrations to show how encapsulation works. By aligning regulatory compliance with robust safety assessment and proactive consumer engagement, soft gel technologies can gain not only market approval but also lasting public confidence, ensuring their role as a safe and innovative component of the modern food industry.

## 6. Conclusions

Over the past few decades, soft gels have transitioned from being perceived as simple gelling agents to becoming multifunctional materials with an active role in modern food systems. They now serve not only as structural components but also as vehicles for targeted nutrient delivery, fat replacement, product preservation, and even active packaging. Their ability to adapt to environmental and physiological stimuli, coupled with their capacity to encapsulate a wide range of bioactive compounds, positions them at the forefront of innovations in functional foods and sustainable packaging.

Our review highlights that hydrogels and organogels have already secured a firm place in commercial applications, supported by their biocompatibility, tunable properties, and established regulatory acceptance. In contrast, aerogels and bigels remain less explored, largely because their production involves high costs, limited mechanical robustness, and complex formulations. New frontiers, including nano-enabled gels and 3D-printed food structures, demonstrate remarkable potential for customization and functionality, yet their path to the market is slowed by safety validation requirements, consumer skepticism, and challenges in scaling up production.

For these technologies to realize their full potential, research must address several urgent priorities. These include developing soft gel systems that can withstand real-world processing, transport, and storage without losing their functional properties; conducting long-term safety assessments using advanced in vitro digestion models and chronic exposure studies; and reducing production costs, especially for aerogels, through energy-efficient manufacturing methods. Sustainability must be embedded into these developments, from the circular sourcing of raw materials to minimizing energy use and conducting life cycle assessments to measure environmental impacts.

From an industrial perspective, the next steps should involve building consumer trust through transparent labeling and participatory communication strategies that demystify new food technologies. At the same time, innovations in manufacturing, such as integrating additive manufacturing with conventional gelation techniques, could provide industries with the precision, flexibility, and scalability needed for market success.

Finally, soft gels are more than passive carriers; they are strategic enablers of solutions to pressing global challenges in health, nutrition, and sustainability. Progress in this field will depend on cross-disciplinary collaboration that unites scientific innovation with regulatory compliance, consumer expectations, and industrial feasibility, ensuring that the next generation of soft gels moves from promising concepts to transformative realities in the food industry.

## Figures and Tables

**Table 1:** Different gel types, examples of use, advantages, and disadvantages.

Gel	Gel Type	Examples of Use	Types of Texture	Advantages	Disadvantages	References
Gelatin Gel	Hydrogel	Gummy vitamins	Elastic, soft	Good encapsulation, widely used	Animal-derived, heat-sensitive	[30]
Pectin Gel	Hydrogel	Fruit jelly, vegan gummies	Soft, melts in mouth	Vegan, fruit-compatible	pH and calcium-sensitive	[31]
Agar Gel	Hydrogel	Asian jelly desserts	Firm, brittle	Stable at room temp.	Brittle texture	[32]
Carrageenan Gel	Hydrogel	Dairy-based desserts	Elastic, creamy	Plant-based, good gelling	Interactions with ions	[33]
Starch Gel	Hydrogel	Candy, jellybeans	Chewy, dense	Cost-effective	Low thermal resistance	[34]
Gelatin-Starch Blend	Hydrogel	Gummy bears	Chewy, resilient	Texture customization	Still animal-derived	[35]
Pullulan/HPMC Gel	Other (polysaccharide film)	Vegan soft capsules	Smooth, soft shell	Vegan, clean label	Expensive	[36]
Alginate Gel	Hydrogel	Spherification, filled candies	Soft outer, liquid core	Unique mouthfeel	Calcium-sensitive	[32]
Gum Arabic Gel	Hydrogel	Flavor encapsulation	Film-like	Natural emulsifier	Low strength	[33]
Monoglyceride Organogel	Organogel	Margarine, shortening	Creamy, firm	Replaces saturated fats; stable	High melting point, slow digestion	[37]
Phytosterol Oleogels	Organogel	Functional spreads, cholesterol-lowering foods	Smooth, semi-solid	Lowers LDL cholesterol, plant-based	Limited thermal stability	[38]
Beeswax Organogel	Organogel	Fat replacer in bakery and meat products	Firm, brittle	Natural, stable, widely available	Brittle at low temp.	[39]
Rice Bran Wax Oleogel	Organogel	Low-fat cookies, meat analogs	Waxy, spreadable	Vegan, sustainable, neutral flavor	Requires precise processing	[40]
Ethylcellulose Organogel	Organogel	Controlled lipid delivery in processed food	Elastic, spreadable	High oil-binding capacity	Needs high temp. to dissolve EC in oil	[41]
Lecithin-Based Organogel	Organogel	Nutrient carriers in oil-rich foods	Fluid–gel	Food-grade emulsifier, bioavailable	Weak mechanical stability	[42,43]
Sorbitan Monostearate Gel	Organogel	Confectionery fat substitutes	Smooth, semi-solid	Food-safe, good structuring agent	Not vegan, limited consumer appeal	[44,45,46]
Apple Pectin Aerogel	Aerogel	Encapsulation of bioactives, food foams	Light, crispy	Biodegradable, high porosity	Fragile under humidity	[47,48]
Cellulose-Based Aerogel	Aerogel	Oil absorption in low-fat frying	Dry, fibrous	High oil retention capacity, biodegradable	Expensive processing	[49,50,51,52]
Starch-Based Aerogel	Aerogel	Flavor and nutrient delivery systems	Porous, melts in mouth	Low-cost, digestible	Less thermal stability	[53]
Chitosan Aerogel	Aerogel	Controlled release in functional food	Lightweight, porous	Antimicrobial, excellent carrier	Allergen potential	[54]
Carrageenan Aerogel	Aerogel	Bioactive compound carrier in beverages	Brittle, friable	Water-soluble, renewable marine source	Sensitive to moisture	[55,56]
Monoglyceride–Beeswax Oleogel and High Acyl Gellan Gum Hydrogel	Bigel	Lycopene delivery	Semi-solid	High encapsulation ability	pH sensitive	[57]
Lecithin, Stearic and Whey Protein Bigel	Bigel	Probiotic survival	Semi-solid	Enhance probiotic resistance	Enzyme sensitive	[58]
Sunflower Oil and Xanthan–Guar Gum Mixture Bigels	Bigel	Ascorbic acid delivery	Semi-solid	High encapsulation efficiency	Less thermal stability	[59]

**Table 2:** Regulatory frameworks and key safety evaluation steps for soft gels in food applications.

Jurisdiction	Food Additive Approval	Food-Contact Material Regulation	Regulatory Body	Key Safety Assessment Steps	References
European Union (EU)	Regulation (EC) No. 1333/2008	Regulation (EU) No. 10/2011	EFSA, European Commission	Toxicological testing; migration limits; long-term exposure studies; bioaccumulation risk assessment; post-market monitoring	[150,151]
United States (USA)	GRAS list (21 CFR Parts 170–186)	FDA Food Contact Notifications (FCNs)	U.S. FDA	GRAS determination; chronic toxicity evaluation; interaction testing with food matrices; surveillance for adverse effects	[152,153]
Canada	Lists of Permitted Food Additives	Food and Drug Act and Regulations	Health Canada	Maximum permissible levels; degradation product safety; cumulative exposure analysis; consumer safety reporting	[154,155]
Australia/New Zealand	FSANZ Food Standards Code	FSANZ Standard 1.4.3	FSANZ	Composition compliance; stability under processing/storage; bioavailability and metabolism studies; continuous compliance audits	[156]
Japan	Food Sanitation Act	Positive List System for food-contact materials	MHLW	Migration testing; biodegradability evaluation; chronic and reproductive toxicity tests; periodic re-evaluation of new materials	[157,158]
India	Food Safety and Standards (Food Product Standards and Food Additives) Regulations, 2011 (and compendium updates)	Food Safety and Standards (Packaging) Regulations, 2018 (Version II 2022); updates to plastic migration limits	FSSAI	Additive permissions by category; migration limits for packaging; toxicology; labeling and surveillance	[159,160]
China	GB 2760 National Food Safety Standard—Standard for Uses of Food Additives (updated; replacing GB 2760-2014)	GB 4806 series for FCMs (material-specific requirements)	NHC (with CFSA), SAMR	Lists of approved or conditionally permitted items; usage categories and scopes; migration considerations and exposure levels; Specific Migration Limits (SMLs) for individual products	[161,162]
Brazil	ANVISA consolidated additive framework (e.g., RDC 778/2023 and amendments), aligned with MERCOSUR	RDC 326/2019 (positive list for additives in food-contact plastics and coatings); transposition of MERCOSUR GMC resolutions	ANVISA	Compliance with positive lists; SMLs such as phthalates; interaction testing; national implementation of GMC regulations	[163,164]

## Data Availability

No new data were created or analyzed in this study. Data sharing is not applicable to this article.
